# Frailty assessment and risk prediction by GRACE score in older patients with acute myocardial infarction

**DOI:** 10.1186/s12877-020-1500-9

**Published:** 2020-03-13

**Authors:** Atul Anand, Sarah Cudmore, Shirley Robertson, Jacqueline Stephen, Kristin Haga, Christopher J. Weir, Scott A. Murray, Kirsty Boyd, Julian Gunn, Javaid Iqbal, Alasdair MacLullich, Susan D. Shenkin, Keith A. A. Fox, Nicholas Mills, Martin A. Denvir

**Affiliations:** 1 BHF Centre for Cardiovascular Science, University of Edinburgh, Room SU.305 Chancellor’s Building, Edinburgh, EH16 4SB UK; 2 Geriatric Medicine Research Group, University of Edinburgh, Edinburgh, UK; 3 Department of Cardiology, Edinburgh Heart Centre, Royal Infirmary of Edinburgh, Edinburgh, UK; 4 Edinburgh Clinical Trials Unit, Usher Institute for Population Health Sciences and Informatics University of Edinburgh, Edinburgh, UK; 5 Primary Palliative Care Research Group, Usher Institute for Population Health Sciences and Informatics, University of Edinburgh, Edinburgh, UK; 6 South Yorkshire Cardiothoracic Centre, Northern General Hospital, Sheffield, UK

**Keywords:** Frailty, Risk prediction, Myocardial infarction, Acute coronary syndrome

## Abstract

**Background:**

Risk prediction after myocardial infarction is often complex in older patients. The Global Registry of Acute Coronary Events (GRACE) model includes clinical parameters and age, but not frailty. We hypothesised that frailty would enhance the prognostic properties of GRACE.

**Methods:**

We performed a prospective observational cohort study in two independent cardiology units: the Royal Infirmary of Edinburgh, UK (primary cohort) and the South Yorkshire Cardiothoracic Centre, Sheffield, UK (external validation). The study sample included 198 patients ≥65 years old hospitalised with type 1 myocardial infarction (primary cohort) and 96 patients ≥65 years old undergoing cardiac catheterisation for myocardial infarction (external validation). Frailty was assessed using the Clinical Frailty Scale (CFS). The GRACE 2.0 estimated risk of 12-month mortality, Charlson comorbidity index and Karnofsky disability scale were also determined for each patient.

**Results:**

Forty (20%) patients were frail (CFS ≥5). These individuals had greater comorbidity, functional impairment and a higher risk of death at 12 months (49% vs. 9% in non-frail patients, *p* < 0.001). The hazard of 12-month all-cause mortality nearly doubled *per* point increase in CFS after adjustment for age, sex and comorbidity (Hazard Ratio [HR] 1.90, 95% CI 1.47–2.44, *p* < 0.001). The CFS had good discrimination for mortality by Receiver Operating Characteristic (ROC) curve analysis (Area Under the Curve [AUC] 0.81, 95% CI 0.72–0.89) and enhanced the GRACE estimate (AUC 0.86 vs. 0.80 without CFS, *p* = 0.04). At existing GRACE thresholds, the CFS resulted in a Net Reclassification Improvement (NRI) of 0.44 (95% CI 0.28–0.60, *p* < 0.001), largely through reductions in risk estimates amongst non-frail patients. Similar findings were observed in the external validation cohort (NRI 0.46, 95% CI 0.23–0.69, *p* < 0.001).

**Conclusions:**

The GRACE score overestimated mortality risk after myocardial infarction in these cohorts of older patients. The CFS is a simple guided frailty tool that may enhance prediction in this setting. These findings merit evaluation in larger cohorts of unselected patients.

**Trial registration:**

Clinicaltrials.gov; NCT02302014 (November 26th 2014, retrospectively registered).

## Background

The population admitted to hospital following myocardial infarction is ageing. Advances in patient care have reduced age-specific mortality rates in developed countries, but this effect is offset by an expanding older population [1, 2]. However, clinical trials in acute coronary syndrome have consistently failed to represent older adults, limiting the generalizability of findings to this age group [3]. The Global Registry of Acute Coronary Events (GRACE) sought to provide a larger and more representative sample, demonstrating significant disparities in the management and in-hospital outcomes for the oldest patients with myocardial infarction [4, 5]. These data have generated GRACE risk estimates which include age amongst other clinical parameters to predict outcomes following myocardial infarction [6, 7].

However, it is recognized increasingly that frailty, as a metric of depleted physiological reserves, better reflects biological age in older adults [8]. Frailty is three-fold more common in older people with cardiovascular disease [9] and these individuals experience double the mortality risk of fitter people independent of age or comorbidity [10]. For risk prediction, gait speed has been shown to add value to Framingham risk scores of patients with ST-segment elevation myocardial infarction [11]. Similarly in patients with non-ST-segment elevation infarctions, the physical frailty phenotype independently predicted major cardiovascular events beyond the GRACE score in the TRILOGY ACS randomised control trial comparing antiplatelet strategies [12]. Similar reports of the effect of frailty on GRACE risk estimates come from studies using physical measures that necessitate additional patient testing [13].

The Clinical Frailty Scale (CFS) is a brief guided tool to assess frailty in hospital settings without specialist equipment [14]. It has been widely used to identify older patients at risk of poorer outcomes [15]. Although it does not require additional equipment or physical measures, the CFS is a precise frailty tool, using specific descriptors of patient symptoms and activity. Our objective was to test the performance of the CFS in an older population with myocardial infarction. We hypothesised that addition of this simple frailty measure would improve the prognostic properties of the GRACE score.

## Methods

### Study design and participants

Patients aged ≥65 years old were assessed prospectively within the screening registry of a phase II randomized controlled trial of future care planning in advanced heart disease at the Royal Infirmary of Edinburgh between October 2013 and September 2014 (NCT 02302014) [16]. Patients with moderate/severe dementia or other barriers to informed consent were excluded. All patients in the registry with a clinical diagnosis of type 1 myocardial infarction were included, except the fifty patients who underwent a tailored intervention in the phase II study. The registry protocol was approved by a local research ethics committee (reference 12/SS/0223) and the study was conducted in collaboration with a UKCRC (UK Clinical Research Collaboration) registered Clinical Trials Unit.

### Frailty measure

The CFS is a structured scale of descriptors to guide selection between nine levels ranging from “very fit: 1” to “terminally ill: 9”. Frailty may be assessed as a continuum, but is considered present at a score ≥ 5 (Supplementary Figure 1) [14]. Assessment criteria include activity, symptoms and assistance usually required with personal activities of daily living (e.g. washing and toileting), and instrumental tasks necessary for independent community living (e.g. managing finances and medications). Clinical nursing staff completed the CFS for each study patient based on their professional assessment and any documentation of premorbid functional status. Nurses received training in the use of the tool from the research team.

### GRACE risk estimate

The GRACE estimated risk of 12-month mortality after myocardial infarction was determined using the online version 2.0 calculator (http://www.gracescore.org/website/WebVersion.aspx) [7]. Clinical and biochemical measures required for the score (heart rate, systolic blood pressure, Killip class, creatinine, cardiac troponin and electrocardiogram changes) were taken from the time of initial presentation with cardiac symptoms. The tool assigns categories based on the calculated risk: low (< 4% estimated 12-month mortality risk), medium (4–12%) and high (> 12%) risk.

### Comorbidity and functional measures

Comorbidity was measured using the Charlson comorbidity index, with a higher score indicating greater comorbidity [17]. Functional status and disability were recorded on the Karnofsky scale, with increasing dependency indicated by a *lower* score in the range of 0–100 [18]. Research staff completed these scales using all available paper and electronic health records (TrakCare; InterSystems Corporation, Cambridge, MA, USA) together with patient or family history.

### Outcomes

Electronic health records were used to determine the primary endpoint of all-cause mortality in the 12 months following index admission. Secondary outcomes were length of index hospital stay, completion of cardiac catheterization (with or without percutaneous coronary intervention), hospital readmissions within 12 months and attendance at cardiac rehabililation.

### External validation cohort

Findings from the primary analysis were also tested in an independent cohort, comprising 96 patients aged ≥65 years old undergoing cardiac catheterization following myocardial infarction at the South Yorkshire Cardiothoracic Centre (Sheffield, UK), a tertiary referral centre for a population of 1.8 million people in the North of England. GRACE and CFS scores were available for all participants. The recruitment and data collection within this cohort has been previously described in detail [19, 20].

### Statistical analysis

Continuous data are presented as means ± SD or median ± IQR and where appropriate compared by Student’s t-test, Mann-Whitney U-test or Analysis of Variance (ANOVA). Categorical data are presented as absolute numbers and percentages and compared by Chi-squared test. Logistic, linear and Cox proportional hazards regression modelling were used to determine predictors of the primary and secondary outcomes. Differences between frailty groups in survival analysis were assessed by log rank test. Receiver operating characteristic (ROC) curve analysis was performed by standard methods for discrimination of 12-month mortality. Model fit was assessed by Akaike and Bayesian Information Criteria (AIC and BIC respectively). Coefficients derived from a multiple logistic regression model including both GRACE and CFS scores from the study population were applied to the exernal validation cohort.

To calculate Net Reclassification Improvement (NRI), each patient was assigned one of three risk categories from the GRACE calculator output (low, medium or high risk). Using the multiple logistic regression model including both GRACE and CFS, all patients were reclassified and reported against the same GRACE risk thresholds. The analysis was performed separately in those who died and survived to assess the net reclassification of patients, thereby accounting for both appropriate and inappropriate reclassifications. This was calculated as an unweighted or ‘dimensionless NRI’. All analyses were completed with R (version 3.3.3). NRI calculations were completed using the pROC package [21].

## Results

The study population comprised 198 patients with type 1 myocardial infarction, the majority of whom were male (58%) and with a mean age of 79 ± 6 years. Baseline characteristics are shown in Table 1. The external validation cohort consisted of 96 patients (61% male, mean age 74 ± 6 years, Supplementary Table 1). Baseline measures and follow-up to 12 months was completed in all patients.

**Table 1:** Baseline characteristics by frailty status

	All patients *(n = 198)*	Not frail (CFS 1–4) *(n = 158)*	Frail (CFS 5–9) *(n = 40)*
Age, years (mean, SD)	79 (6)	78 (6)	83 (7)
Male	115 (58)	99 (63)	16 (40)
Primary diagnosis			
Non-ST segment elevation myocardial infarction	151 (76)	122 (77)	29 (73)
ST-segment elevation myocardial infarction	47 (24)	36 (23)	11 (28)
Past medical history			
Heart failure	36 (18)	21 (13)	15 (38)
Stroke	35 (18)	25 (16)	10 (25)
Diabetes mellitus	35 (18)	32 (20)	3 (8)
Chronic kidney disease (stage III or worse)	45 (23)	30 (19)	15 (38)
Peripheral vascular disease	28 (14)	21 (13)	7 (18)
Chronic respiratory disease	39 (20)	25 (16)	14 (35)
Dementia	14 (7)	7 (4)	7 (18)
Current or ex-smoker	118 (60)	96 (61)	22 (55)
Medications at recruitment			
Total number prescribed drugs (mean, SD)	9.2 (3.1)	8.9 (3.1)	10.5 (2.9)
Aspirin	178 (90)	144 (91)	34 (85)
Clopidogrel	183 (92)	145 (92)	38 (95)
ACE-inhibitor or ARB	127 (64)	113 (72)	14 (35)
Diuretic	80 (40)	57 (36)	23 (58)
Beta-blocker	118 (60)	98 (62)	20 (50)
Statin	155 (78)	132 (84)	23 (58)
Oral anticoagulant	26 (13)	22 (14)	4 (10)
Laboratory measures			
Haemoglobin, g/L (mean, SD)	13.1 (1.8)	13.1 (1.8)	12.7 (2.0)
Creatinine, μmol/L (mean, SD)	104 (45)	101 (39)	119 (62)
Estimated GFR, ml/min/1.73 m2 (mean, SD)	62 (24)	65 (23)	54 (24)
Risk and functional measures			
GRACE 2 12-month mortality (mean, SD)	18.5 (13.2)	16.2 (11.7)	27.8 (15.0)
Charlson comorbidity index (mean, SD)	2.9 (1.8)	2.6 (1.6)	3.9 (2.2)
Karnofsky scale score (mean, SD)	78.5 (17.1)	84.6 (11.8)	54.5 (13.0)

Values are number (%) unless specified

Not frail defined by CFS 1–4 (very fit, well, managing well, vulnerable) and frail by CFS 5–9 (mildly frail, moderately frail, severely frail, very severely frail, terminally ill)

Abbreviations: *CFS* Clinical Frailty Scale, *GFR* glomerular filtration rate (calculated by the Modification of Diet in Renal Disease equation), *ACE* angiotensin converting enzyme, *ARB* angiotensin receptor blocker, *GRACE* Global Registry for Acute Coronary Events

### The CFS identifies a high-risk group of patients with poorer outcomes

**Fig. 1:**
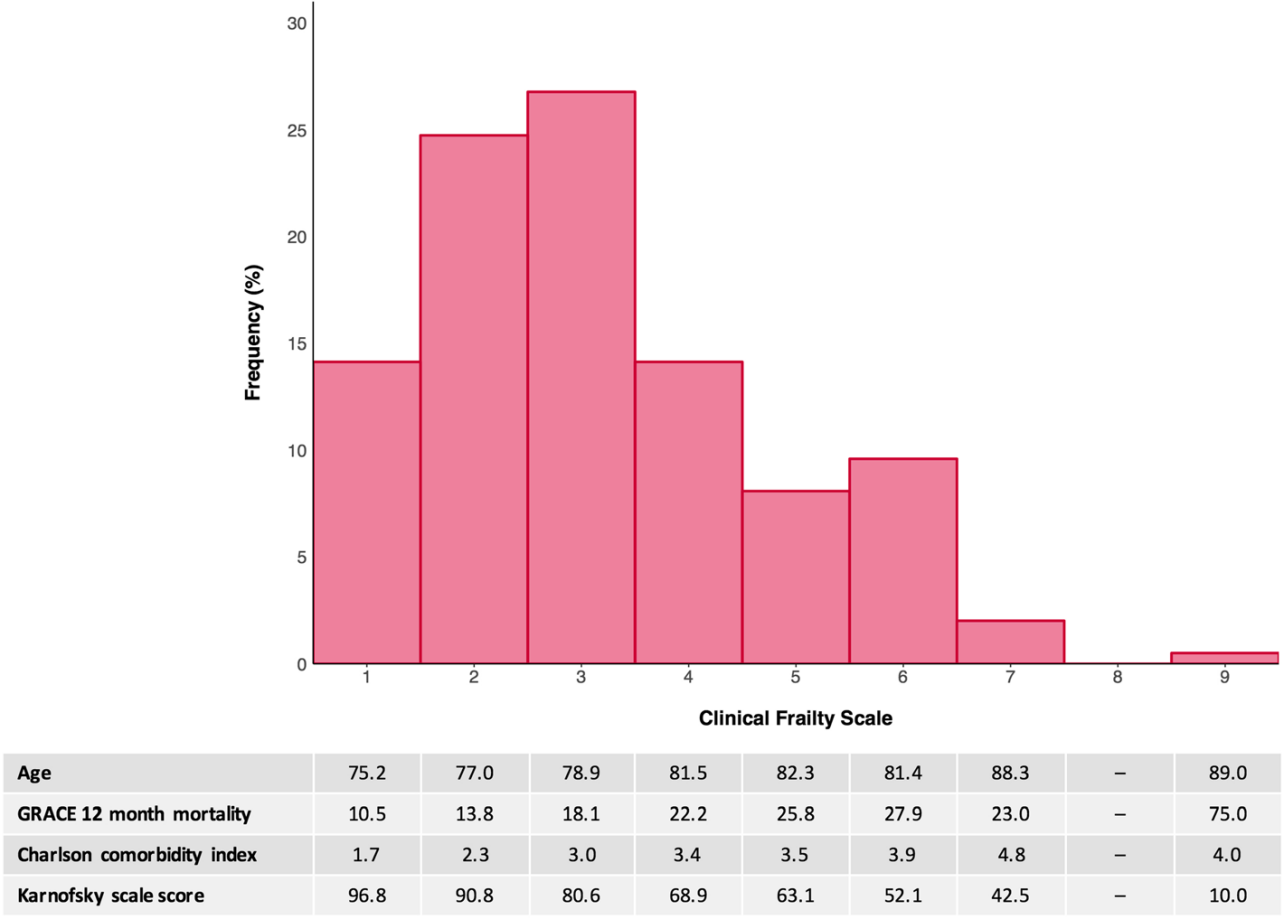
Frequency histogram for the distribution of Clinical Frailty Scale scores (range 1–9) in the study population. Appended table shows the mean age, GRACE 12-month mortality risk estimation, Charlson comorbidity index and Karnofsky scale score in the study sample with each CFS score. By ANOVA, each of these measures demonstrated a significant change with increasing CFS scores (all *p* < 0.001)

The CFS identified 40 (20%) patients with frailty defined by a CFS score ≥ 5 (Fig. 1). Using this established CFS threshold, frail patients were older, more often female and experienced greater comorbidity (mean Charlson Comorbidity Index 3.9 ± 2.2 vs. 2.6 ± 1.6 in those with CFS ≤4, *p* < 0.001, Table 1). There were notably higher levels of previous heart failure, chronic kidney disease, respiratory illness and dementia amongst frail patients. Age, comorbidity, functional impairment and GRACE estimated mortality risk increased with higher CFS scores (*p* < 0.001 for all, Fig. 1). Overall, GRACE estimated 12-month mortality was greater in frail patients (28% vs. 16% in those with CFS ≤4, *p* < 0.001).

Frail patients were prescribed more medications before hospital admission (10.5 ± 2.9 vs. 8.9 ± 3.1 with CFS ≤4, *p* = 0.003). Index hospitalization was also longer (11 days [IQR 5–24] vs. 4 days [IQR 3–7] with CFS ≤4, *p* < 0.001), but cardiac catheterization was undertaken less frequently in frail patients (33% vs. 81% with CFS ≤4, *p* < 0.001, Table 2). Observed mortality was higher in frail patients, with 15% dying during the index hospitalization and nearly half by 12 months (48% vs. 9% with CFS ≤4, *p* < 0.001). Thus, frailty was associated with spending a shorter period alive and out of hospital in the year following index hospitalization (mean 229 ± 137 vs. 329 ± 68 days with CFS ≤4, *p* < 0.001). In those that survived to hospital discharge, no patient with CFS ≥5 attended cardiac rehabilitation. In non-frail patients, each point increase in CFS was associated with a reduced likelihood of attendance at rehabiliation, after adjustment for age, sex and baseline comorbidity (adjusted odds ratio [OR] 0.59, 95% confidence intervals [CI] 0.42–0.80 per unit increase in CFS, *p* = 0.001). However, the CFS did not predict hospital readmissions over the 12 months after index admission following adjustment (adjusted HR 1.10 (0.86–1.40), *p* = 0.44).

**Table 2:** Outcomes by frailty from multivariate regression modelling

	All patients *(n = 198)*	Not frail (CFS 1–4) *(n = 158)*	Frail (CFS 5–9) *(n = 40)*	Unadjusted RR per unit increase in CFS	*p*-value	Adjusted RR per unit increase in CFS^a^	*p*-value
Index hospital admission outcome							
Cardiac catheterization	141 (71)	128 (81)	13 (33)	0.46 (0.35–0.58)	< 0.001	0.53 (0.40–0.70)	< 0.001
Dead	10 (5)	4 (3)	6 (15)	1.98 (1.60–2.44)	< 0.001	1.90 (1.47–2.44)	< 0.001
At 12 months							
Attended cardiac rehabilitation^b^	62 (33)	62 (40)	0 (0)	0.49 (0.36–0.64)	< 0.001	0.59 (0.42–0.80)	0.001
Readmitted^b^	46 (24)	34 (22)	12 (35)	1.39 (1.17–1.67)	< 0.001	1.10 (0.86–1.40)	0.44
Dead	33 (17)	14 (9)	19 (48)	1.98 (1.60–2.44)	< 0.001	1.90 (1.47–2.44)	< 0.001

Outcomes presented by frailty status as numbers (%). Modelling is Cox proportional hazards for death and readmission (producing a hazard ratio), and logistic regression for cardiac catheterization and cardiac rehabilitation (producing an odds ratio). In all cases, frailty is considered as a continuous variable. Each risk ratio (hazard ratio or odds ratio) presented as risk change per unit increase in CFS (from 1 to 9) with 95% confidence intervals

^a^Adjusted for age, sex and Charlson Comorbidity Index score

^b^In those who survived to discharge (*n* = 188; not frail *n* = 154; frail *n* = 34)

### The CFS is an independent predictor of mortality risk

Considering frailty as a continuous variable, each point increase in CFS score predicted a doubling of the hazard of 12-month mortality (HR 1.98, 95% CI 1.60–2.44, *p* < 0.001), with the effect minimally attenuated after adjustment for age, sex and comorbidity (adjusted HR 1.90, 95% CI 1.47–2.44, *p* < 0.001, Table 2). The relationship between CFS and mortality remained independent following adjustment for the GRACE score (adjusted HR 1.72, 95% CI 1.37–2.16 per unit increase in CFS, *p* < 0.001). Similarly the Karnofsky score remained an independent predictor of mortality beyond GRACE (adjusted HR 0.96, 95% CI 0.94–0.98 per unit increase, *p* < 0.001), but the Charlson comorbidity index did not (adjusted HR 1.21, 95% CI 0.99–1.46, *p* = 0.06). By linear regression modelling, the number of days alive and out of hospital in the 12 months after index admission decreased by 25 days with each point increase in CFS (95% CI 18–33 days, *p* < 0.001).

**Fig. 2:**
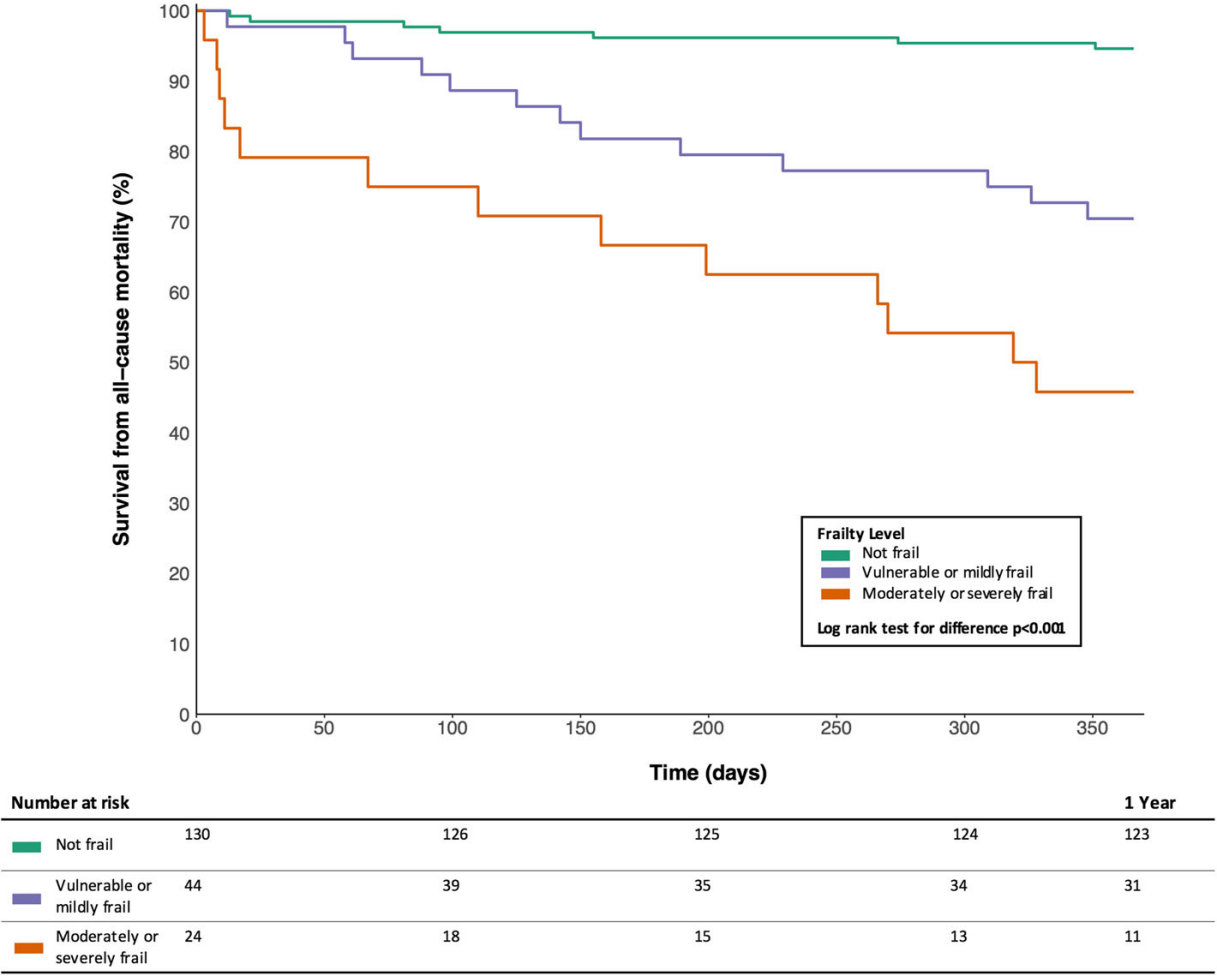
Kaplan-Meier survival plot for the year following index hospital admission, stratified by frailty status. Frailty defined by CFS thresholds for not frail (CFS 1–3), vulnerable or mild frailty (CFS 4–5) and moderate to severe frailty (CFS 5–9). Log rank test for difference *p* < 0.001

Using the CFS to divide the population into non-frail (CFS 1–3), vulnerable or mildly frail (CFS 4–5) and moderate to severely frail (CFS 6–9) patients demonstrated separation of survival curves throughout the 12 months following index hospitalization (log rank test *p* < 0.001, Fig. 2).

### Addition of the CFS improves discrimination of the GRACE risk estimate

**Fig. 3:**
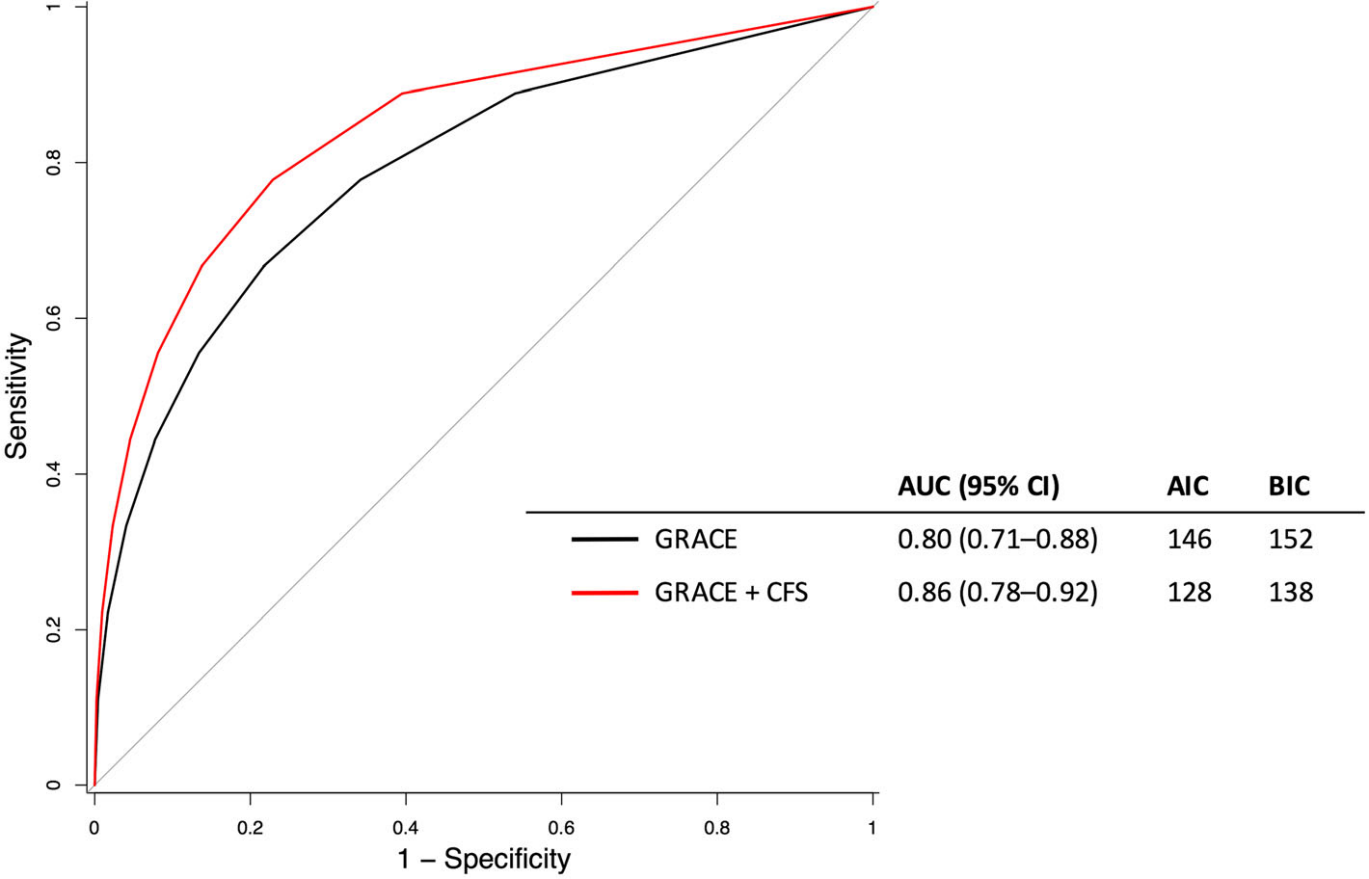
ROC curve for the prediction of 12-month mortality by GRACE score and with addition of CFS in multiple logistic regression modelling. AUC = area under the curve; AIK = Akaike Information Criteria; BIC = Bayesian Information Criteria

The area under the ROC curve (AUC) for an outcome of 12-month mortality after myocardial infarction was 0.80 (95% CI 0.71–0.88) for the GRACE score and 0.81 (95% CI 0.72–0.89) for the CFS. These were stronger discriminators than either the Charlson comorbidity index (AUC 0.68, 95% CI 0.58–0.78) or Karnofsy score (AUC 0.76, 95% CI 0.68–0.87, Supplementary Figure 2). Addition of the CFS to GRACE improved the AUC to 0.86 (95% CI 0.78–0.92), which represented a significant gain by DeLong testing (*p* = 0.04, Fig. 3). Both the AIC and BIC were lower in the model including the CFS score implying improved model fit.

### Addition of CFS to GRACE results in net reclassification improvement

By GRACE estimation, high-risk status (> 12% risk of 12-month mortality) was applied to 112 (57%) patients, medium risk (4–12% risk) to 81 (41%) patients and low risk (< 4% risk) to 5 (3%) patients. Within the high-risk cohort, survival curves demonstrated continued separation by frailty status (log-rank test *p* < 0.001, Supplementary Figure 3). Addition of the CFS in a multiple logistic regression model with the GRACE score resulted in a NRI of 0.44 (95% CI 0.28–0.60, *p* < 0.001), with the majority of reclassification events downgrading estimated risk (Supplementary Table 2). Amongst all patients who were still alive at 12 months, 97 (59%) moved to a lower GRACE risk category with the updated model.

### Expected and observed mortality

Across the study population, the predicted 12-month mortality by GRACE estimate was 18.5%, which compared to an observed mortality of 16.7%. Dividing the population by CFS frailty status identified an overestimation of risk amongst patients with CFS ≤4 (16.2% predicted vs. 8.9% observed), and an underestimation in frail patients (27.8% predicted vs. 47.5% observed with CFS ≥ 5, Supplementary Table 3). Formal calibration testing was not performed due to sample size.

### External validation

In the external validation cohort, 27 (28%) patients were frail (CFS ≥5). There were 14 (15%) deaths within 12 months. After applying the multiple logistic regression model coefficients derived in the main study to this external validation cohort, the AUC for 12-month mortality was 0.75 (95% CI 0.60–0.87), although this was not a significant improvement on GRACE alone (*p* = 0.40, Supplementary Figure 4). By NRI, applying the main study model improved classification in the validation cohort (0.46, 95% CI 0.23–0.69, *p* < 0.001), once more through reductions in estimated risk using the model including CFS (Supplementary Table 4).

## Discussion

We have studied the utility of a simple frailty measure for risk prediction in older patients after myocardial infarction and have made a number of important observations. First, the GRACE tool overestimated 12-month mortality risk in our study population. Second, a guided frailty tool could be incorporated into routine clinical care to identify patients at high risk of poor outcomes. Third, the Clinical Frailty Scale was an independent predictor of mortality in an older myocardial infarction population and added significant discrimation to the GRACE 12-month mortality estimate. Finally, frailty reclassified mortality risk in nearly half of this population, largely through identification of older but fitter individuals. This simple measure of frailty has potential to improve risk assessment in the large population of older patients recovering from myocardial infarction.

There are a number of strengths to our study. Frailty assessment was performed by clinical nursing staff using existing clinical data and without any specialist equipment. This approach would appear feasible for inclusion into the routine care of older cardiology patients based on our experience in a busy tertiary referral centre. In contrast to other studies [22, 23], we have not focussed solely on short-term risk estimation and have directly assessed frailty against an existing and widely used clinical tool. Further, we have undertaken external validation suggesting wider applicability of these findings, although this would benefit from larger validation studies. With advances in cardiac care, the majority of even the oldest patients survive an acute infarct [24]. The attention of risk prediction after myocardial infarction has therefore shifted from immediate survival to recovery, rehabilitation and future risk-stratification. Identification of vulnerability to poor outcomes could guide tailored cardiac rehabilitation, follow-up and future intervention strategies.

It may be surprising that most reclassification gains occurred by downgrading the estimated GRACE risk in robust patients. This challenges preconceptions that frailty assessment only adds value in those nearing death; resilience in fitter older patients may be equally informative. However, at the other end of this spectrum, it is striking that half of the patients identified as frail by CFS assessment had died within 12 months of myocardial infarction. Frailty assessment offers the potential for future care planning in this targeted population, which we have previously shown to be feasible and acceptable in our phase II study [16]. Individualized decision-making including frailty could therefore increase clinician confidence in the management of patients across the range of vulnerability from low to high risk. Such an approach is the antithesis of ageism, and may assist in targeting increasingly complex interventions towards to those most likely to experience benefit [25].

Our findings may reflect excessive reliance on age in current risk determination. At a population level there is no doubt that ageing increases the risk of almost all harmful outcomes, but chronological age fails to capture individual differences. At its core, frailty exposes the variation in ageing trajectories between individuals [8]. In this study, age was not independently predictive of any outcome once frailty was included in modelling. It is however important to recognize that the GRACE estimation, which includes age as a key variable, peformed well in the discrimination of 12-month mortality in the study population, but worth acknowledging that frailty could improve this further and provide net reclassification benefit. Such improvements in the discrimination of existing risk scores are rarely achieved by new biomarkers [26] and this finding merits further evaluation in larger cohorts, or perhaps in future iterations of the GRACE tool.

The 2015 European Society of Cardiology guidelines recommend use of the GRACE 2.0 calculator to assess patient risk after myocardial infarction, stating that the value of such tools is “undisputed”. However, the guidelines acknowledge that “the impact of risk score implementation on patient outcomes has not been adequately investigated” [27]. The complexities of managing frail patients are recognized, particularly with regard to invasive strategies and adjusted dose regimes for antiplatelets, beta-blockers and ACE-inhibitors. In our study no frail patients attended cardiac rehabilitation, in keeping with referral patterns observed elsewhere [28]. This is despite increasing evidence in favour of structured physical activity programmes amongst individuals living with frailty [29]. The European Association of Preventative Cardiology have recently identified the pressing clinical need for further research into the area of frailty and cardiac rehabilitation [30]. No specific frailty tool is recommended by these guidelines, which in part reflects a lack of consensus in the broader frailty literature. A recent systematic review identified reports of 67 different frailty tools, of which the CFS is one of the most highly cited [15].

Other recent guidelines for the management for older patients have focussed on multimorbidity, such as from the National Institute for Health and Care Excellence (NICE) [31]. However, the Charlson comorbidity index did not add to the GRACE estimation in our study, suggesting that simplistic counts of comorbidity may be less important than assessing the functional manifestations of these conditions. We have demonstrated that the Karnofsky performance scale was an independent predictor of mortality beyond GRACE, although discrimination was not as strong as with the CFS. It is possible that such functional and disability scales may add further objectivity to the classification of frailty.

Our study has some limitations. The study population was recruited for potential selection into a study of future care planning. An age cutoff was chosen to enrich for the frailty measure, but this limited the identification of younger patients with impairments. The study cohort was therefore likely to have been at higher risk than a general, older cardiology population. Despite this, only 20% of our study population were graded with a CFS score ≥ 5, which represents a realistic proportion for additional intervention such as future care planning. The CFS did not independently predict hospital readmission, but this may be related to competing mortality risk amongst frail patients. It is critical that an effective frailty measure does not saturate in the target population, as this would lack any clinical utility beyond age. The distribution of CFS scores across our study was significantly less frail than in the Canadian older community-dwelling population in which it was first described [14]. This may be due to a younger but more medically comorbid sample hospitalized with myocardial infarction, but may also reflect our limited sample size. Although we undertook external validation this was also performed in a small cohort.

## Conclusions

In conclusion we have demonstrated the effectiveness of a simple frailty tool in the assessment of older patients after myocardial infarction. The CFS could be included in the routine clinical assessment of cardiology patients, providing improved discrimination of the existing GRACE estimate for 12-month mortality. Risk assessment including frailty has potential to enhance individualized decisions in cardiac patients. These findings merit evaluation in larger and unselected cohorts.

## Supplementary information

**Additional file 1.** Supplementary material including 4 additional data tables and 4 additional figures as referenced in the results section.

## Data Availability

The datasets generated and/or analysed during the current study are not publicly available due the specifics of the consent obtained from individual participants, but are available from the corresponding author on reasonable request.

## Abbreviations

AIC: Akaike Information Criterion
ANOVA: Analysis of Variance
AUC: Area Under the Curve
BIC: Bayesian information criterion
CFS: Clinical Frality Scale
GRACE: Global Registry of Acute Coronary Events
NICE: National Institute for Health and Care Excellence
NRI: Net Reclassification Index
OR: Odds Ratio
ROC: Receiver Operating Characteristics
UKCRC: United Kingdom Clinical Research Collaboration
