# Supplementary material for: Frailty assessment and risk prediction by GRACE score in older patients with acute myocardial infarction
Source: BMC Geriatr. 2020 Mar 13;20:102. doi: 10.1186/s12877-020-1500-9 (PMC7069195; doi:10.1186/s12877-020-1500-9)
Supplement: Supplementary file 1 — Additional file 1. Supplementary material including 4 additional data tables and 4 additional figures as referenced in the results section. [file 12877_2020_1500_MOESM1_ESM.docx]

SUPPLEMENTARY MATERIAL

**Frailty and risk prediction by GRACE score in older patients**

**with myocardial infarction**

Atul Anand, Sarah Cudmore, Shirley Robertson, Jacqueline Stephen, Kristin Haga, Christopher J. Weir, Scott A. Murray, Kirsty Boyd, Julian Gunn, Javaid Iqbal, Alasdair MacLullich, Susan D. Shenkin, Keith A.A. Fox, Nicholas Mills, Martin A. Denvir

**Supplementary Table 1: Baseline characteristics of the external validation cohort**

|  | All patients  *(n=96)* | Not frail  (CFS 1-4)  *(n=69)* | Frail  (CFS 5-9)  *(n=27)* | |
| --- | --- | --- | --- | --- |
| Age, years (mean, SD) | 74 (6) | 72 (6) | 78 (5) | |
| Male | 59 (62) | 48 (70) | 11 (41) | |
|  |  |  |  | |
| *Past medical history* |  |  |  | |
| Stroke | 5 (5) | 2 (3) | 3 (11) | |
| Diabetes mellitus | 27 (28) | 12 (17) | 15 (56) | |
| Peripheral vascular disease | 30 (31) | 21 (30) | 9 (33) | |
| Chronic respiratory disease | 14 (15) | 9 (13) | 5 (19) | |
| Dementia | 4 (4) | 0 (0) | 4 (15) | |
|  |  |  |  | |
| *Medications at recruitment (prior to index MI)* |  |  |  | |
| Aspirin | 37 (39) | 24 (35) | 13 (48) | |
| Dual antiplatelet | 20 (21) | 13 (19) | 7 (26) | |
| ACE-inhibitor or ARB | 38 (40) | 24 (35) | 14 (52) | |
| Beta-blocker | 38 (40) | 24 (35) | 14 (52) | |
| Statin | 44 (46) | 29 (42) | 15 (56) | |
|  |  |  |  | |
| *Risk measure* |  |  |  | |
| GRACE 12-month mortality estimate (mean, SD) | 16.9 (19.6) | 13.2 (14.6) | 26.5 (26.6) | |
| Values are number (%) unless specified  Abbreviations: ACE = angiotensin converting enzyme; ARB = angiotensin receptor blocker; GRACE = Global Registry for Acute Coronary Events.  Not frail defined by CFS 1-4 (very fit, well, managing well, vulnerable) and frail by CFS 5-9 (mildly frail, moderately frail, severely frail, very severely frail, terminally ill) | | | |  |

**Supplementary Table 2: Net reclassification events in the study population.** Cells contain the number of patients in each risk strata. Green cells represent appropriate reclassification events (i.e. move to a lower risk strata in survivors, or a higher strata in those that died). Red cells represent inappropriate reclassifications (i.e. move to a higher risk strata in those that survived or lower risk in those that died).

***Alive at 12 months***

|  |  | **GRACE + CFS** | | |
| --- | --- | --- | --- | --- |
|  |  | Low (<4%) | Medium (4–12%) | High (>12%) |
| **GRACE** | Low (<4%) | 5 | 0 | 0 |
|  | Medium (4–12%) | 53 | 20 | 4 |
|  | High (>12%) | 14 | 30 | 39 |

***Dead at 12 months***

|  |  | **GRACE + CFS** | | |
| --- | --- | --- | --- | --- |
|  |  | Low (<4%) | Medium (4–12%) | High (>12%) |
| **GRACE** | Low (<4%) | 0 | 0 | 0 |
|  | Medium (4–12%) | 1 | 2 | 1 |
|  | High (>12%) | 0 | 4 | 25 |

**Supplementary Table 3: Expected versus observed mortality**

|  | 12 month mortality | |  |
| --- | --- | --- | --- |
|  | Expected | Observed |  |
| All patients | 18.5 | 16.7 |  |
|  |  |  |  |
| Not Frail (CFS 1-4) | 16.2 | 8.9 |  |
|  |  |  |  |
| Frail (CFS 5-9) | 27.8 | 47.5 |  |
| Values are the percentage mortality at 12 months either observed in the study population or expected from calculation of GRACE estimate. | | | |

**Supplementary Table 4: Net reclassification events in the external validation cohort.** Cells contain the number of patients in each risk strata. Green cells represent appropriate reclassification events (i.e. move to a lower risk strata in survivors, or a higher strata in those that died). Red cells represent inappropriate reclassifications (i.e. move to a higher risk strata in those that survived or lower risk in those that died).

***Alive at 12 months***

|  |  | **GRACE + CFS** | | |
| --- | --- | --- | --- | --- |
|  |  | Low (<4%) | Medium (4–12%) | High (>12%) |
| **GRACE** | Low (<4%) | 5 | 2 | 0 |
|  | Medium (4–12%) | 23 | 14 | 8 |
|  | High (>12%) | 3 | 10 | 17 |

***Dead at 12 months***

|  |  | **GRACE + CFS** | | |
| --- | --- | --- | --- | --- |
|  |  | Low (<4%) | Medium (4–12%) | High (>12%) |
| **GRACE** | Low (<4%) | 0 | 0 | 0 |
|  | Medium (4–12%) | 0 | 4 | 2 |
|  | High (>12%) | 0 | 0 | 8 |

**Supplementary Figure 1: Clinical Frailty Scale guide**

**
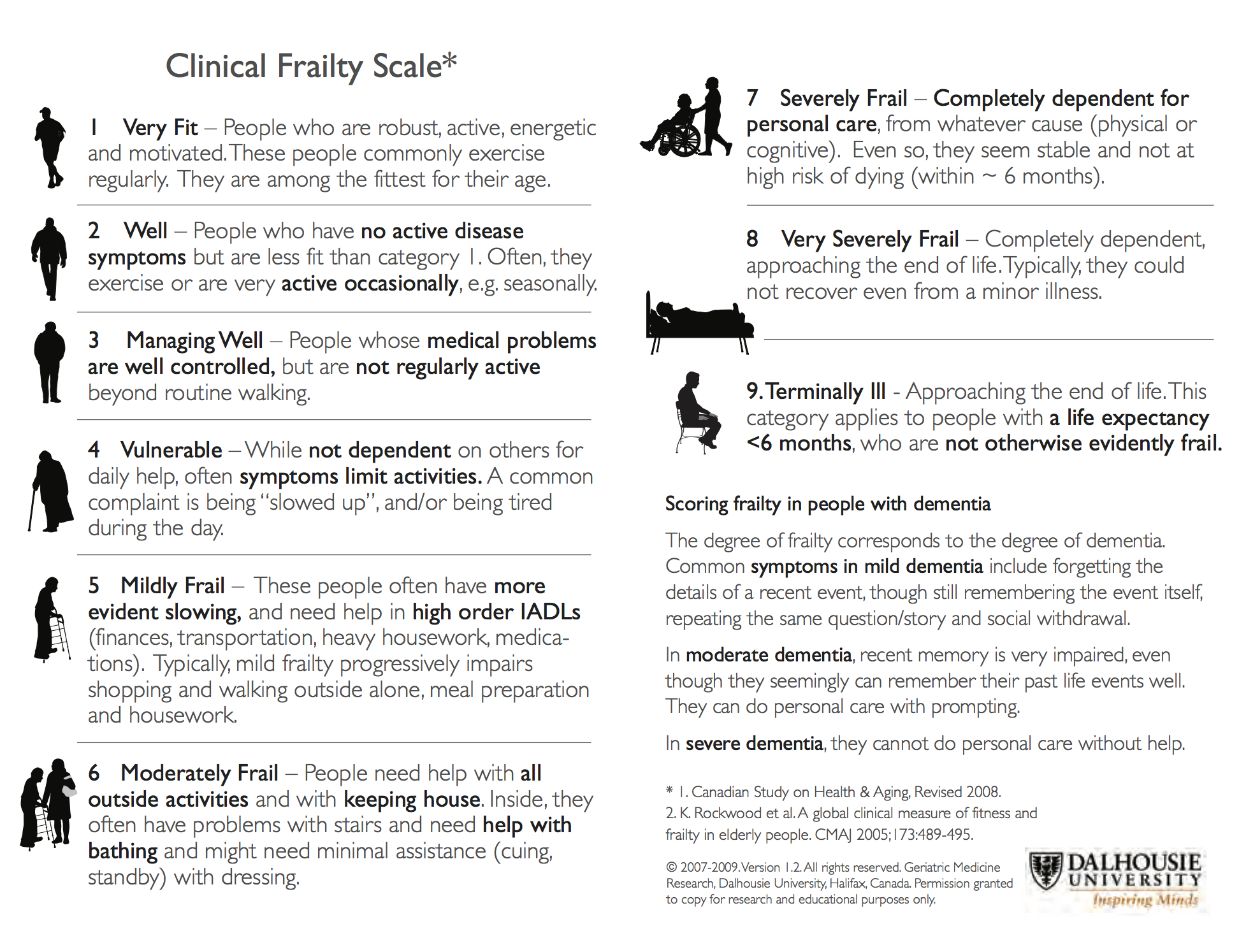
**

**Supplementary Figure 2: ROC curve analysis for the prediction of 12 month mortality by CFS, Karnofsky and Charlson scores**

**
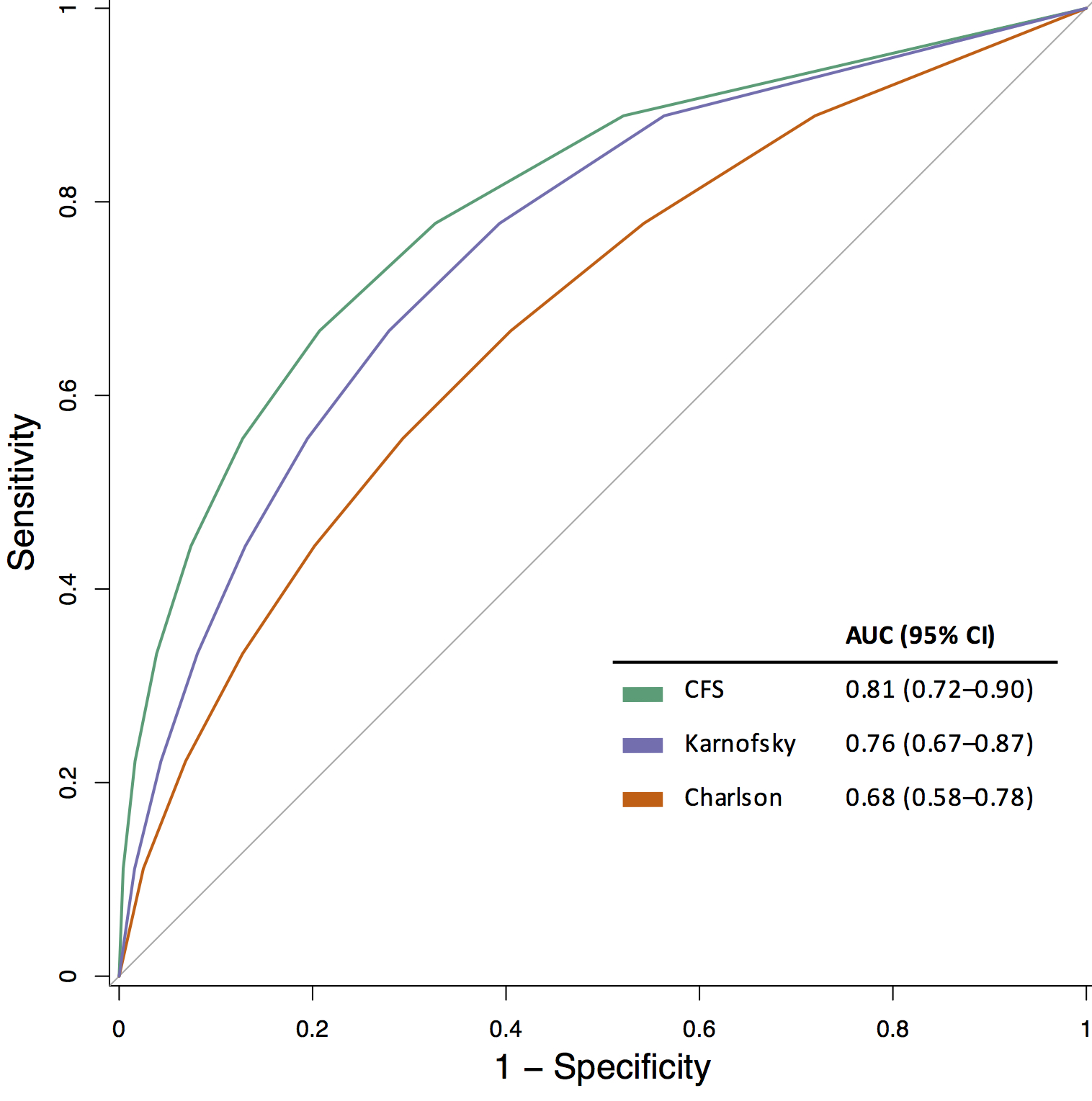
**

**Supplementary Figure 3: Separation of survival by frailty status amongst high-risk GRACE patients (estimated 12 month mortality >12%)**

**
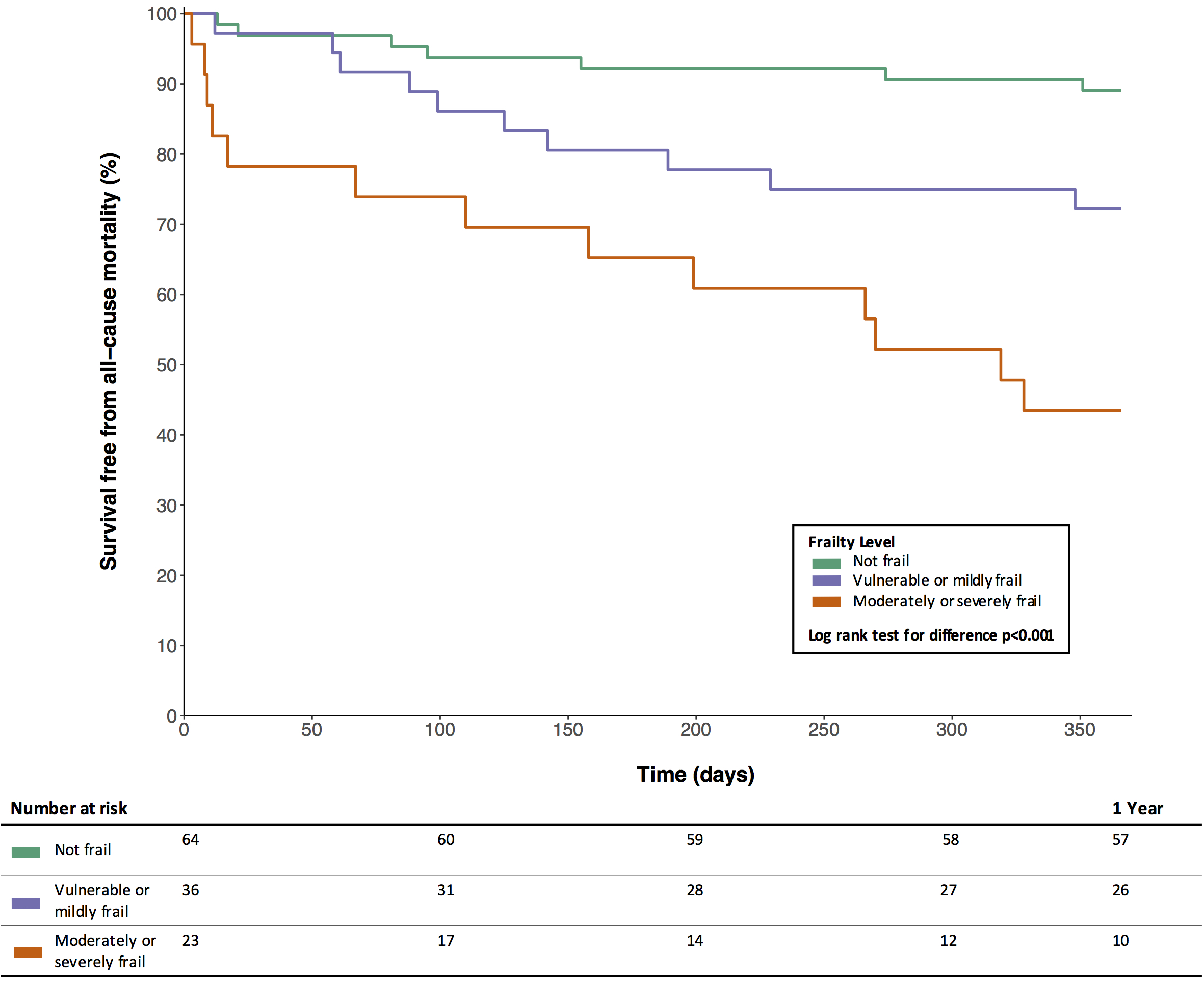
**

**Supplementary Figure 4: ROC curve analysis for the prediction of 12 month mortality by GRACE and updated GRACE + CFS model in the external validation cohort**

**
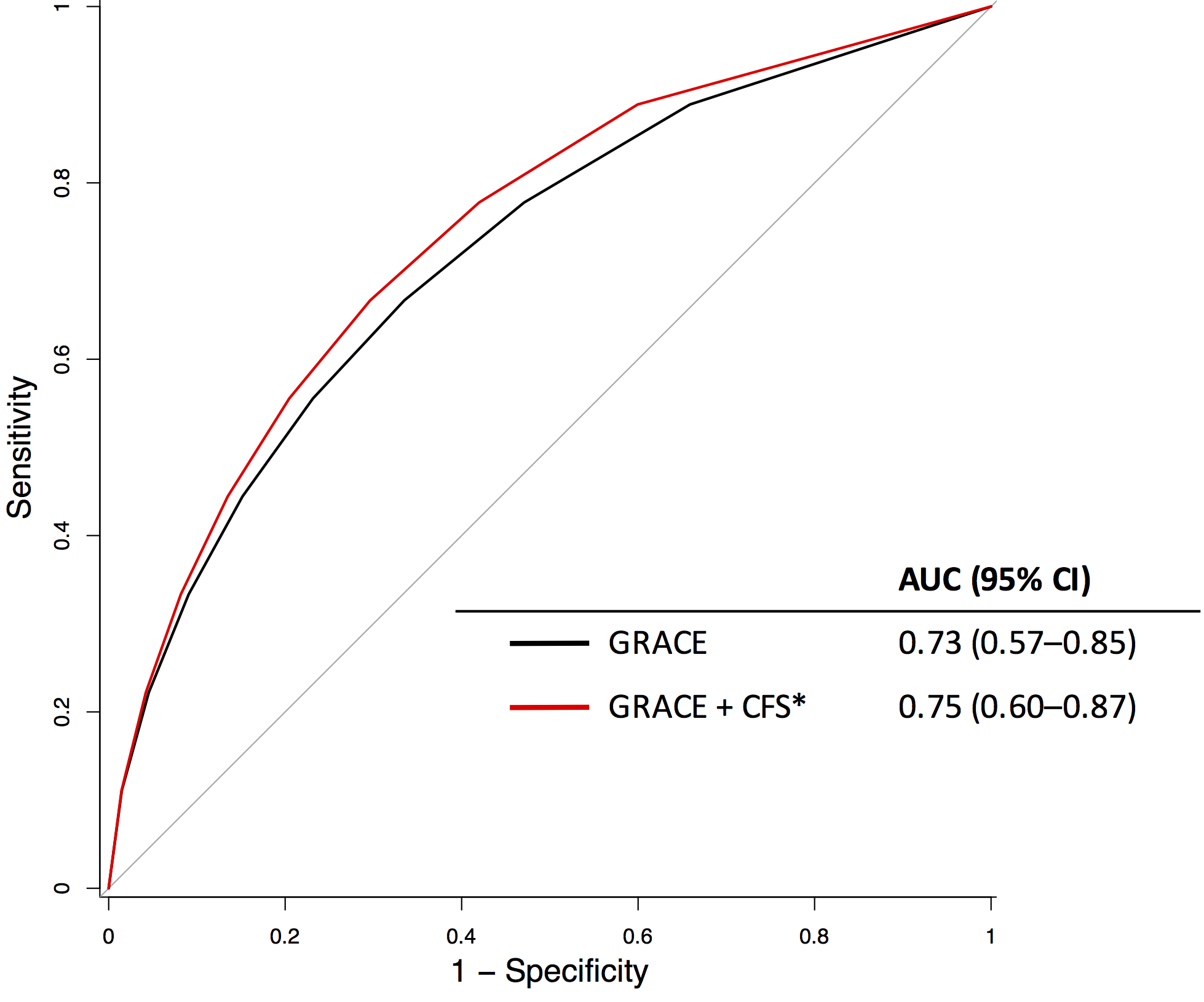
**

*Using model derived in larger study dataset applied to the external validation cohort
